# Persistent Postoperative Hiccups

**DOI:** 10.1155/2020/8867431

**Published:** 2020-07-04

**Authors:** Emily Bryer, Jeffrey Bryer

**Affiliations:** ^1^Department of Internal Medicine, Pennsylvania Hospital, Pennsylvania, PA, USA; ^2^West Chester Psychiatric Associates, West Chester, PA, USA

## Abstract

Hiccups are a common and poorly understood pathologic phenomenon. While hiccups often occur suddenly and episodically, they may persist for weeks and sometimes months. There is a paucity of data regarding the precise etiology and optimal treatment for persistent hiccups. Frequently considered a benign and frustrating condition, hiccups are sometimes a presenting symptom for pulmonary embolism and cardiac disease. We present a patient with gastroesophageal reflux disease who developed 11 days of recurrent hiccups following an orthopedic procedure.

## 1. Case Description

A healthy 68-year-old Caucasian male with a past medical history significant for gout, asthma, and gastroesophageal reflux disease presented for evaluation of subacute right posterior tibial tendon dysfunction. All vital signs were stable on arrival, and all labs were normal. His medications were famotidine 10 mg (recently changed from omeprazole 40 mg), allopurinol 100 mg, and rosuvastatin 10 mg. He drank one glass of wine every evening and had no tobacco or drug use. EKG showed normal sinus rhythm at a regular rate without any evidence of ischemia or infarction. Chest X-ray was without any active cardiopulmonary disease. The patient underwent general endotracheal anesthesia with propofol along with a right popliteal nerve block prior to tibial tendon transfer, medializing calcaneal osteotomy, and posterior tibial tendon reconstruction. He received standard of care intraoperative cephalosporin, and there were no surgical or airway complications. The patient was discharged home with a recommendation to avoid weight-bearing status and to take aspirin 162 mg daily.

On postoperative day 1, the patient developed hiccups as described by a sudden diaphragmatic contraction coupled with vocalization. Following the initial vocalization, the patient progressed to silent diaphragmatic contractions up to 9-10 in a row that persisted for 10 seconds (Video 1). This precluded respiration, swallowing, and speaking; this continued for hours at a time with contractions of increasing frequency. He developed prevomiting salivation which often prefaced the end of a course of contractions; however, at times, actual vomiting occurred. There was no temporal involvement to the occurance of hiccups in relation to food.

The following medications were trialed without successfully terminating contractions: metoclopramide 10 mg every 6 hours, chlorpromazine 25 mg three times daily, baclofen 10 mg twice daily, clonazepam 0.5 mg as needed, and gabapentin 300 mg three times daily. On day 8 of persistent hiccups, the patient went to the emergency department for evaluation. EKG and all labs, including troponin, were normal. A computed tomography of the chest was obtained and excluded a pulmonary embolism. He was given metoclopramide and aluminum/magnesium hydroxide suspension and discharged home without relief. The hiccups continued for a total of 9 days prompting pulmonary consultation. The patient was then started on gabapentin 600 mg three times daily and omeprazole 40 mg two times daily. One complete day on this regimen resulted in complete cessation of hiccups. He completed a slow taper of gabapentin and omeprazole and was continued on famotidine 10 mg daily monotherapy.

## 2. Review

Hiccups are referred to as both synchronous diaphragmatic flutter and singultus. They result from a sudden reflexive spasmodic contraction of the diaphragm that precedes sudden closure of the glottis with corresponding vocalization. The hiccup process occurs over 35 milliseconds [1]. The mammalian hiccup reflex is achieved via afferent pathways (phrenic nerve, vagus nerve, or thoracic sympathetic fibers from T6–T10), central processor (medulla oblongata), and an efferent pathway (phrenic nerve) [2]. Any physical, chemical, inflammatory, or neoplastic irritant that affects a component of this reflex arc may induce hiccups [3]. Hiccups are categorized by their duration: transient (seconds to minutes); persistent (48 hours–1 month); and intractable (greater than one month) [2, 4]. Recurrent hiccups refer to repeated episodes exceeding a few minutes [2].

**Figure 1:**
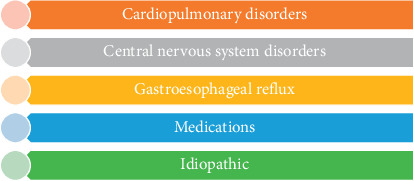
Etiology of hiccups.

Although there is no universally recognized etiology of hiccups, there are a variety of hypotheses related to their origin. From a Darwinian perspective, the burping reflex signifies a survival advantage as young mammals who depend on milk for their nutrition need to displace swallowed air in the abdomen from continuous suckling in order to make room for more milk [5]. Hiccups are often associated with specific medications and conditions (Figure 1). Some of these medications include dopaminergic agonists which potentiate hiccups via affinity for the D3 receptor, as illustrated by 20% of Parkinsonism patients with hiccups [6]. Consequently, dopamine antagonists are often used in the treatment of hiccups including metoclopramide and chlorpromazine [6]. Other frequently implicated medications include dexamethasone, azithromycin, benzodiazepines, and propofol [7]. Patients who experience hiccups with dexamethasone usually cease when transitioned to methylprednisolone [8, 9]. One possible mechanism of steroids prompting or perpetuating hiccups includes a decrease in the threshold for synaptic transmission in the midbrain [9]. A variety of chemotherapy drugs also may cause hiccups including levofolinate, fluorouracil, oxaliplatin, carboplatin, and irinotecan [10–13]. Hiccups have been reported in a variety of central nervous system disorders including ischemic, vascular, neoplastic, and structural lesions. They are a frequent symptom of lateral medullary infarction also known as “Wallenberg syndrome.” [14]

While transient hiccups are commonly of unclear etiology, persistent hiccups often result from gastroesophageal dysfunction and disease [2]. Despite gastroesophageal disease as an etiology of hiccups, it is interestingly also a complication of recurrent hiccups [2]. In normal clinical practice, recurrent hiccups are not frequently encountered, nor do many physicians consider themselves well versed in diaphragmatic conditions such as hiccups. Given the rarity of recurrent hiccups in clinical practice and the resulting lack of physician treatment, this condition is often considered relatively benign and of brief and self-limiting duration. However, hiccups may be the only presenting symptom of cardiopulmonary disease. Inferior wall myocardial ischemia, pericarditis, and pulmonary emboli may stimulate and irritate the phrenic nerve, resulting in hiccups [2, 3, 7]. Although the precise mechanism remains unknown, some research studies suggest that pulmonary emboli may irritate either the afferent or efferent arms of the hiccup reflex arc [7].

As cardiac ischemia and pulmonary embolism are potentially life-threatening clinical conditions, persistent and intractable hiccups necessitate further investigation of a potentially lethal origin. Further investigation may include a thorough history, physical, labs, and imaging studies. Frequent lack of definitive etiological understanding in presentations of hiccups leads to considerable variation in treatment approaches. Although not validated in randomized controlled trials, physical maneuvers to terminate hiccups may be successful if the hiccups last for less than 48 hours [4]. Some of these techniques for vagal stimulation include breath holding, Valsalva maneuvers, pressing on the eyeballs, sipping cold water, and pulling on the tongue [4].

**Figure 2:**
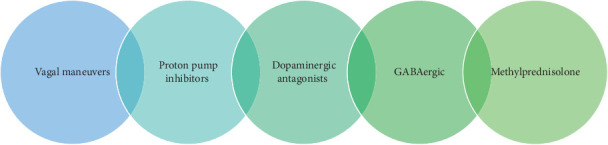
Treatment of hiccups.

In addition to physical maneuvers, pharmacologic treatment, presented in order of escalatory therapy, is indicated if the hiccups persist for more than 48 hours (Figure 2). Most of these therapies involve drugs that affect dopaminergic and or GABAergic pathways [15]. Selection of a pharmacologic therapy often involves the exclusion of reversible causes. When gastroesophageal reflux is considered a possible causative factor, a trial of a proton pump inhibitor may be initiated. If not efficacious or if gastric disease is not a likely offender, gabapentin, baclofen, and metoclopramide are reasonable first-line agents [4]. Drugs that affect GABAergic pathways, such as gabapentin and benzodiazepines, mitigate hiccups through the inhibition of voltage-operated calcium channels and subsequent release of neurotransmitters, glutamate and substance P, to modulate the diaphragmatic activity [16–18].

There are limited data to support the use of second-line agents including anticonvulsants, antidepressants, antiarrhythmics, and central nervous system stimulants [4]. If pharmacotyerapy escalation and combination do not relieve symptoms, other hiccup therapies include acupuncture, hypnotherapy and diaphragm-related interventions such as phrenic nerve stimulation [4, 19].

## 3. Case Discussion

Although hiccups can be a transient and benign entity, the persistent duration of hiccups in this case for 11 days postoperatively is peculiar. Persistent hiccups in the postoperative setting are an underreported and important phenomenon to both recognize and investigate. This patient had risk factors for venous thromboembolic disease after recent orthopedic operation and subsequent immobility and endothelial injury. After pulmonary embolism and cardiac ischemia were excluded, other, less critical, triggers were explored. Development of recurrent hiccups in this current patient was likely multifactorial given the history of gastroesophageal reflux disease and recent discontinuation of omeprazole. Although the use of propofol carries a rate of hiccups <1% [20], it is possible that anesthesia may have contributed to hiccups and functional gastroparesis [21, 22]. Substituting famotidine for omeprazole in the months leading up to the surgery may have resulted in decreasing acid suppression corresponded with worsening GERD which resulted in hiccups. This hypothesis is supported by cessation of symptoms with initiation of omeprazole 40 twice daily. However, this is confounded by the coinitiation of gabapentin 600 mg three times daily which may have led to hiccup cessation via stimulation of GABAergic pathways.

## 4. Conclusion

Hiccups are a common and frequently transient nuisance. In rare cases, recurrent or intractable hiccups may signify a potentially life-threatening cardiopulmonary condition and require clinical evaluation. In addition to a thorough history and physical exam, labs, imaging, and further diagnostic tests may be indicated to investigate the origin. Vagal stimulation as well as therapies targeting dopaminergic and GABAergic pathways may provide relief from persistent hiccups. While hiccups can be a benign entity, persistent hiccups should prompt evaluation for serious, and potentially fatal, life-threatening conditions.

## Supplementary Materials

Supplementary Materials The attached video demonstrates the persistent hiccups and diaphragmatic contractions described in this case.
